# Reproductive outcomes of 3 infertile males with XYY syndrome: Retrospective case series and literature review

**DOI:** 10.1097/MD.0000000000019375

**Published:** 2020-02-28

**Authors:** Xinyue Zhang, Xiangyin Liu, Qi Xi, Haibo Zhu, Linlin Li, Ruizhi Liu, Yang Yu

**Affiliations:** Center for Reproductive Medicine, Center of Prenatal Diagnosis, First Hospital of Jilin University, Changchun, China.

**Keywords:** assisted, genetic counseling, infertility, reproductive techniques, XYY karyotype

## Abstract

The aim of this study is to evaluate the pregnancy outcomes of males with a 47, XYY karyotype following assisted reproductive treatment.

A retrospective study was performed using data from infertile men with 47, XYY at a center for reproductive medicine in 2004 to 2017. Of the 19,842 infertile males treated, a total of 21 showed the 47, XYY karyotype and were included in the present study. Clinical variables were collected. Three men were under treatment with their partner before either in vitro fertilization (IVF) or intracytoplasmic sperm injection (ICSI).

The incidence of 47, XYY in infertile men is 1/945 (21/19842). Most men are azoospermic or severely oligospermic. Three men and their partners underwent IVF or ICSI treatment with fresh ejaculate samples. The fertilization rate was 52.94% to 83.33%. The embryo cleavage rate was 50% to 90%. One man had abnormal sex hormonal levels and his partner had no clinical pregnancy. The other 2 couples had healthy baby boys.

Live spermatozoa can be gathered and fertility is possible for infertile males with 47, XYY syndrome when IVF or ICSI treatment is used. It is recommended that genetic counseling is provided in such cases.

## 1 Introduction

Sex chromosome aneuploidies (SCA) such as Klinefelter syndrome (47, XXY), Turner syndrome (45, X), 47, XXX and 47, XYY syndromes occur approximately once in every 420 live births.^[1]^ The 47, XYY syndrome is a common sex chromosomal genetic syndrome that occurs approximately once in every 1000 live male births. Because of the diverse phenotype of the syndrome and potential lack of symptoms, identification, and diagnosis of men with 47, XYY syndrome is difficult. Common symptoms include greater risk of behavioral problems, mild learning difficulties, delayed speech and language development, and tall stature.^[3]^ Most males with 47, XYY, approximately 85%, are not diagnosed until they present with fertility problems.^[4]^ This means that the majority of 47, XYY men have a delayed diagnosis, with a median age of 17.1 years at diagnosis.^[2]^ The study reported 47, XYY syndrome occurs more frequent in infertile men. These men have a 4-fold increased infertility risk compared to men with 46, XY.^[5]^

Several studies demonstrated that men with 47, XYY syndrome have variable sperm counts, ranging from normozoospermia to azoospermia^[6,7]^ and increased sperm chromosomal abnormalities.^[8]^ While carriers of the 47, XYY syndrome can produce offspring without medical intervention, even passing the extra Y chromosome to offspring. A few of men with 47, XYY syndrome had difficulty to achieve pregnancy. Assisted reproductive technologies (ART), such as in vitro fertilization (IVF) and intracytoplasmic sperm injection (ICSI) may be required to produce offspring.^[9]^ However, methods for evaluating and treating XYY syndrome have not been established. In this study, the medical records of 21 men with 47, XYY syndrome, and their clinical and laboratory sperm parameters, were investigated. Of these patients, the results of in IVF or ICSI cycles performed in 3 men diagnosed infertility are also reported.

## 2 Materials and methods

### 2.1 Patients

A retrospective study was performed using data from infertile men with 47, XYY at the Center for Reproductive Medicine, First Hospital of Jilin University from 2004 to 2017. The average age of the patients was 28.1 ± 5.2 years (range 21–41 years). Three men were undergoing fertility treatment with their partners, before either IVF or ICSI. A questionnaire, designed to obtain information on smoking history, alcohol intake, working conditions, past medical history, injuries, and any familial conditions, was completed by all subjects. The patients’ gave written informed consent, and the study was approved by the ethics committee of the First Hospital of Jilin University.

### 2.2 Semen analysis

Semen samples were collected by masturbation after 3 to 5 days of sexual abstinence and semen characteristics were detected within 1 hour of ejaculation using computer-assisted semen analysis (http://www.wei-li.com/). Semen analysis was performed according to the World Health Organization standard protocol (fifth edition) (http://www.who.int/en/). Patients were diagnosed with oligozoospermia if their semen samples (taken at intervals of 1–3 weeks) had sperm counts <20 × 10^6^/mL, moderate oligozoospermia if their sperm counts were >5 × 10^6^/mL and <10 × 10^6^/mL, severe oligozoospermia if their sperm counts were <5 × 10^6^/mL, azoospermia if no sperm were present in at least 3 ejaculates after centrifugation and asthenospermia if sperm progressive motility [(a + b)%] was <50%.

### 2.3 Hormonal analysis

Peripheral blood samples were drawn from the majority of patients. Serum was collected from centrifuged blood samples at 1000 g for 15 minutes. The concentrations of plasma follicle-stimulating hormone (FSH), luteinizing hormone (LH), and testosterone (T) were measured using electrochemiluminescence immunoassay (Elecsys 2010 Chemistry Analyser, Mannheim, Germany). Normal reference ranges for these hormones in the male are as follows FSH, 1.5 to 12.4 mIU/mL; LH, 1.7 to 8.6 mIU/mL; and T, 9.9 to 27.8 nmol/mL.

### 2.4 Karyotype analysis

Peripheral blood lymphocytes from all patients were collected and cultured in lymphocyte culture medium (Yishengjun, Guangzhou Baidi Biotech Co. Ltd, China) at 37°C for 72 hours and then treated with colcemid (Sigma, Uk) for 1 hour. Peripheral blood lymphocytes were harvested and processed by hypotenic treatment, fixation, trypsinization, and Giemsa banding (GTG-banding). For each patient, a minimum of 20 metaphase cells were counted, and at least 3 cells were analyzed.

### 2.5 Y chromosome microdeletion analysis

Genomic DNA was isolated from Ethylene Diamine Tetraacetic Acid-treated peripheral blood using a commercially available whole-blood DNA extraction kit (TIAN amp Blood DNA kit, Beijing Tiangen Biotech, Beijing, China). Y chromosome microdeletion analysis was performed by polymerase chain reaction (PCR) amplification of sequence tagged sites (STS) or gene-specific sequences, according to the recommendations of the European Academy of Andrology and the European Molecular Genetics Quality Network. Specific STS markers spanning the entire azoospermia factor region were used: sY84, sY86, sY127, sY134, sY143, sY254, and sY255. ZFX/ZFY loci were used as internal controls. For cases with no band visualized, PCR analysis was performed a minimum of 2 times to verify the deletion.

### 2.6 Ovarian stimulation and ART procedure

Ovarian induction was performed using a gonadotrophin-releasing hormone (GnRH) analog suppression (Tryptorelin, Ferring, Germany) protocol (short or long), a GnRH agonist protocol. Ovarian stimulation was performed as previously reported.^[10]^ Oocyte retrieval was performed 36 to 40 hours after human chorionic gonadotropin (HCG) injection. IVF or ICSI was performed at 40 hours and 42 hours post-hCG, respectively. 16 to 18 hours after IVF or ICSI, the presence of 2 pronuclei (PN) and 2 polar bodies indicated fertilization. The fertilization rate was calculated as the proportion of metaphase II oocytes forming 2 PN. At 72 hours after oocyte retrieval, embryos were classified according to cleavage and morphology score.^[11]^

The embryo cleavage rate was calculated as the percentage of fertilized oocytes that reached the cleavage stage. Two embryos were transferred into the woman's uterine cavity on day 3 after oocyte retrieval or transferred after cryopreservation and a thawing–warming procedure.

## 3 Results

### 3.1 Clinical findings from 21 infertile men

**Figure 1:**
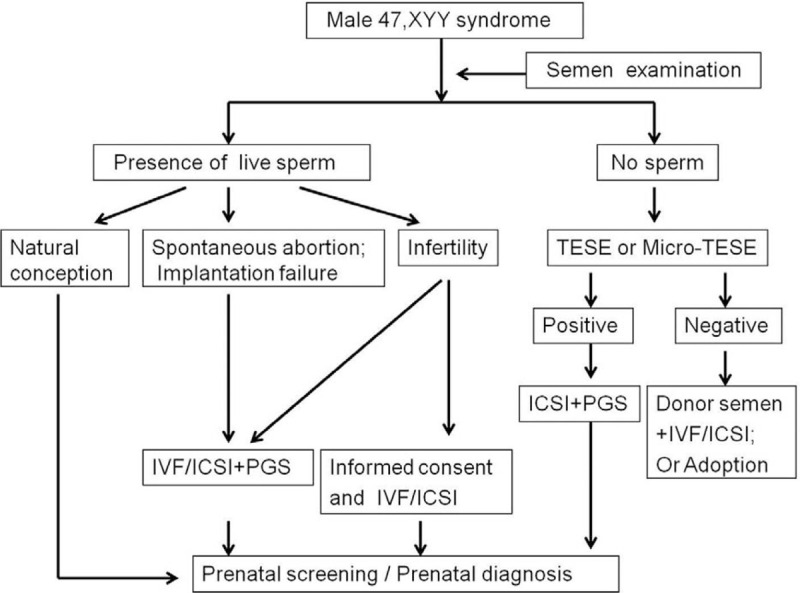
Flow chart of the genetic counseling procedure for a male with 47, XYY syndrome. ICSI = intracytoplasmic sperm injection, IVF = in vitro fertilization, micro-TESE = microdissection testicular sperm extraction, PGS = preimplantation genetic diagnosis for aneuploidy screening, TESE = testicular sperm extraction.

Of the 19,842 infertile males, a total of 21 with karyotype 47, XYY were included in the present study. Therefore, the incidence of 47, XYY in this infertile male population was 1/945 (21/19,842). Semen analysis findings included: 5 cases of azoospermia, 8 cases of severe oligozoospermia (including 5 asthenospermia), 3 cases of moderate oligozoospermia and asthenospermia, 1 case of oligozoospermia and asthenospermia, 2 cases of normozoospermia (including 1 asthenospermia). The results of semen analysis for the other 2 cases were not available (1 performed Y chromosome microdeletions analysis does not absent any STS loci). Y chromosome microdeletion analysis showed that 7 severe oligozoospermia and 4 azoospermia were not deletions of STSs (Table 1). The flow chart of genetic counseling for these males are shown in Figure 1.

**Table 1:**
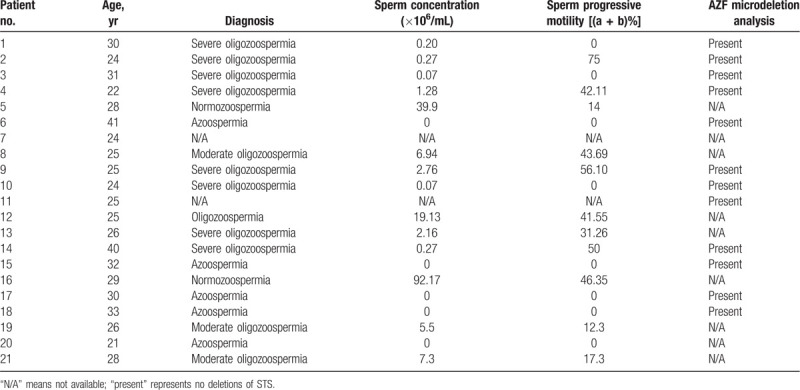
Semen analysis and AZF results of 21 male patients with 47, XYY.

### 3.2 Reproductive hormone assays

Of the 21 infertile men, 10 patients underwent serum sex hormonal analysis. Of these 10 cases, 5 cases had abnormal hormonal level. In these 5 cases, 3 cases (patient numbers 15, 18, 20) had levels of FSH and LH that were higher than normal reference ranges and one of these (patient number 15) also had lower T levels compared with normal reference ranges. Two cases (patient number 6, 11) had higher FSH or higher LH levels and both had lower T levels compared with normal references ranges (Table 2).

**Table 2:**
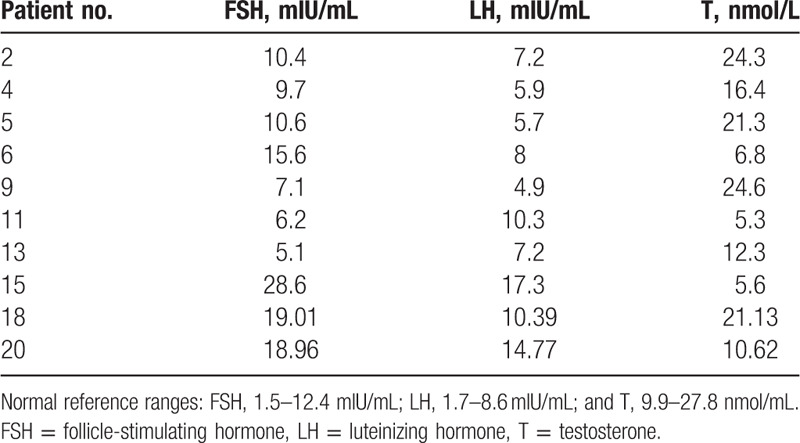
Clinical sex hormonal level of 10 infertile men with 47, XYY.

### 3.3 ART outcomes for 3 infertile males with 47, XYY

Three treatment cycles were performed using fresh ejaculate samples from the 3 male patients (patient number 4, 5, 13). All of the 3 couples had a history of primary infertility. The chromosome karyotype results of the 3 female partners were normal. Female aetiologies of infertility were ovulation dysfunction or polycystic ovary syndrome. Based on the paternal semen concentration, 2 couples performed conventional IVF and the other couple underwent conventional ICSI. Because our clinic was not able to perform preimplantation genetic diagnosis for aneuploidy screening (PGS) procedures they were advised to seek PGS from any authorized provider in China before ART. Nevertheless, for personal reasons these couples chose to undergo IVF/ICSI without PGD.

Clinical outcomes of the ICSI or IVF performed are presented in Table 3. The fertilization rate was 52.94% to 83.33%. The embryo cleavage rate was 50% to 90%. Embryo transfer (ET) was conducted with patient number 5 using 2 fresh embryos (6 IV, 4 IV) and a phenotype normal boy was delivered at 38 weeks gestation. Patient number 4 and number 13 underwent ET with frozen–thawed embryos. Patient number 4 gave birth to a phenotype normal boy at 39 weeks gestation. Genetic information of these born boys showed normal karyotype and no deletions on the Y chromosome.

**Table 3:**
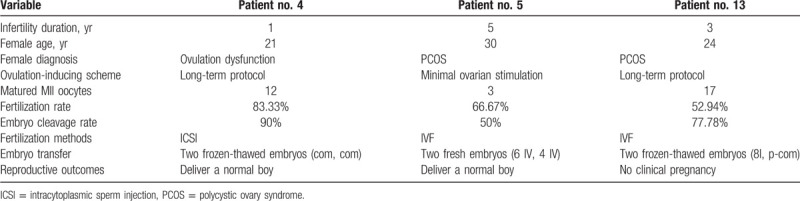
Clinical outcomes of ART for 3 infertile male with 47, XYY.

## 4 Discussion

Nonmosaic 47, XYY syndrome is an aneuploidy of sex chromosomes. It is often associated with male infertility. In our study the incidence rate was 0.1% (21/19,842) which is in accordance with a previously reported incidence rate.^[12]^ Our study on infertile nonmosaic 47, XYY patients confirms that, if spermatozoa can be gathered, fertility is possible with the use of IVF or ICSI treatment.

Individuals with 47, XYY karyotype present with variable phenotypes and the majority of cases are phenotypically normal. About 75% of patients with this syndrome are not prenatally or postnatally detected, and diagnosis is often delayed or absent.^[13,14]^ DNA polymorphism analysis has shown that most cases of SCA occur due to meiotic errors arising in the paternal germline.^[15,16]^ In the case of 47, XYY the syndrome always involves a paternal error as the embryo inherits 2 Y chromosomes. Semen analysis of the patients in this study showed that 47, XYY syndrome carriers can have highly variable sperm counts that range from normal to azoospermia, though most men were azoospermic or severely oligospermic (Table 1), which is in accordance with previous reports (Table 4). There were also reports that males with the 47, XYY karyotype have normal testosterone levels.^[29]^ However, another study found that males in prison had an increased frequency of the 47, XYY karyotype and higher testosterone levels.^[4]^ In our study, 10 patients with XXY syndrome underwent serum sex hormonal analysis and, of these, 5 cases had abnormal hormonal levels (Table 2). Three of the 5 patients had a T level that was lower than normal reference ranges, and all 5 patients had higher FSH and/or LH levels, similar to a previous report.^[26]^ Therefore, abnormal semen analysis results or hormonal level may be an indicator of chromosomal defects in infertile males.

**Table 4:**
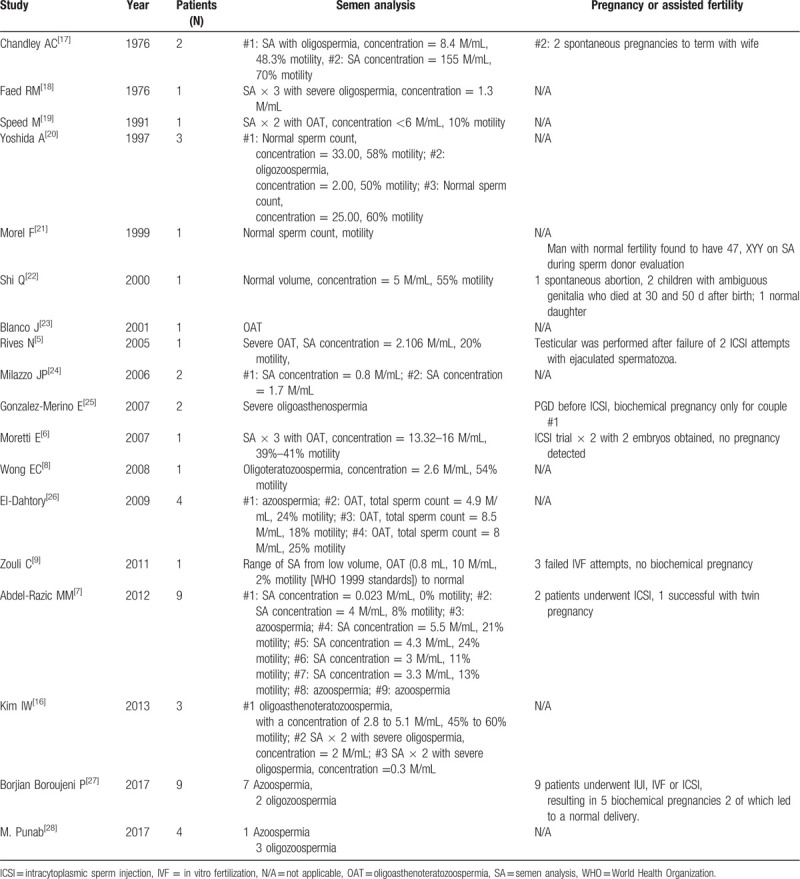
Summary of literature on the semen analysis and reproductive outcomes of men with 47, XYY.

Although fertility may vary in XYY men, the studies have reported an increased incidence of chromosomally abnormal spermatozoa in their semen. Studies had found that XYY infertile men often have an impaired chromosome synapsis and are missing a meiotic recombination site. These errors may make the cells susceptible to meiotic arrest and increase the levels of aneuploidy within spermatozoa.^[30–32]^ If spermatozoa is present, studies have reported a majority of pachytene cells (58%–100%) in the ejaculate of patients that contain the extra Y chromosome.^[8]^ Thus in theory, there is a significant risk of fetal demise and transmission of a SCA in future offspring, at frequency of 50%. However, in clinical practice, the incidence of abnormal karyotype of offspring in published studies is highly variable. In fact, previous studies have reported that descendants of 47, XYY syndrome rarely had chromosomal abnormalities, with an incidence rate of less than 1%.^[22,33]^

The purported mechanism behind this correction is a loss of the extra Y chromosome germ cell during the spermatogonial stages.^[15]^ The study indicated that an arrest point for genetically abnormal germ cells may reside at the primary and secondary spermatocyte or spermatid stages of development leading to a continuous elimination of these cells during spermatogenesis.^[16]^ Solari et al^[34]^ observed a high level of germ cell death at, or immediately after, the meiotic divisions. Milazzo et al^[24]^ also observed a large number of apoptotic round spermatids and impaired meiotic division. Therefore, the presence of the extra Y chromosomes in spermatocytes may cause varying degrees of maturation arrest as well as heterogeneous sperm concentrations.

Men with 47, XYY syndrome that show normal semen parameters can achieve pregnancy spontaneously. However, for infertile men who have difficulty achieving pregnancy, IVF or ICSI may be required. The studies are also demonstrate these findings causing different reproductive outcomes (Table 4). The use of ART can provide an opportunity for these men to father children but the techniques may elevate the risk of conceiving offspring with genetic anomalies.^[35]^ Therefore, genetic counseling is recommended for infertile patients with 47, XYY syndrome. Sperm fluorescence in situ hybridization or PGS can also be considered to better understand the potential risks to the offspring.^[16,25]^ A flow chart of the genetic counseling procedure for these males is shown in Figure 1.

Studies have provided preliminary evidence to suggest higher levels of sperm aneuploidy in 47, XYY men are related to recurrent miscarriage and repetitive ICSI failure.^[30,36]^ In the present study, spermatozoa from three 47, XYY men were used for IVF or ICSI and 2 couples of these patients delivered healthy offspring. The other couple did not have a clinical pregnancy after transfer of 2 frozen–thawed embryos (Table 3). The present study revealed that the fertilization rates of the 3 cases were between 52.94% and 83.33%. This is in accordance with previous reports of 66%^[37]^ and 54.2%,^[38]^ and these results did not show lower fertilization rates as others have reported.^[30]^

In conclusion, men with a 47, XYY karyotype are often phenotypically normal and difficult to diagnose. The methods for precisely evaluating and treating 47, XYY syndrome have not been established. For now, there were no systematic studies have been published showing that how those infertile males with 47, XYY syndrome to fathered a healthy baby, and in such cases, genetic counseling is also recommended.

## Author contributions

Yang Yu and Xinyue Zhang contributed to the design of the article. Xiangyin Liu and Xinyue Zhang contributed to the analysis of data and wrote the manuscript. Qi Xi, Haibo Zhu, and Linlin Li contributed to the collection of data. RuiZhi Liu was the research advisor.
